# The Relationship between Intimacy Change and Passion: A Dyadic Diary Study

**DOI:** 10.3389/fpsyg.2017.02257

**Published:** 2017-12-22

**Authors:** Bülent Aykutoğlu, Ahmet Uysal

**Affiliations:** Department of Psychology, Middle East Technical University, Ankara, Turkey

**Keywords:** intimacy, passion, romantic relationship, diary studies, dyadic data

## Abstract

In the current study we investigated the association between intimacy and passion by testing whether increases in intimacy generates passion (Baumeister and Bratslavsky, 1999). Furthermore, we examined whether there are partner effects in intimacy change and passion link. Couples (*N* = 75) participated in a 14-day long diary study. Dyadic multilevel analyses with residualized intimacy change scores showed that both actors’ and partners’ intimacy change positively predicted actor’s passion. However, analyses also showed that residualized passion change scores positively predicted intimacy. Although these findings provide some empirical evidence for the intimacy change model, in line with the previous research (Rubin and Campbell, 2012), they also suggest that it is not possible to discern whether intimacy increment generates passion or passion increment generates intimacy.

## Introduction

Intimacy and passion are two of the three main components of love (Sternberg, 1986). The link between intimacy and passion has been explored in several studies, and research suggests that these two components of love are closely associated with each other (e.g., Marston et al., 1998; Ng and Cheng, 2010). However, Baumeister and Bratslavsky (1999) proposed a unique theoretical model for the link between intimacy and passion. They proposed that increases in intimacy produce passion, and this association could be moderated by various psychological factors.

Although it has been more than a decade since the model was proposed, the model was not empirically tested until recently (Rubin and Campbell, 2012). In the current research, we conducted a 14-day long diary study to provide a further test of the model and we aimed to replicate the main findings of Rubin and Campbell (2012).

### Intimacy and Passion in Romantic Relationships

Intimacy is defined as “feelings of closeness, connectedness, and bondedness in a close relationship” (Sternberg, 1986). Intimacy has both affective and cognitive elements such as self-disclosure (Clark and Reis, 1988; Baumeister and Bratslavsky, 1999), communication of affection (Sternberg and Grajek, 1984; Baumeister and Bratslavsky, 1999), perceived partner responsiveness (Reis et al., 2004), and positive attitudes toward the partner (Sternberg and Grajek, 1984; Sternberg, 1986; Acker and Davis, 1992). Intimacy accumulates over time as partners become more acquainted with each other and develop a deep understanding of each other, a process in which self-disclosure plays an important role. Consequently, intimacy steadily builds up during the early stages of a relationship and eventually becomes stagnant (Sternberg, 1986).

Passion, on the other hand, is primarily affective. Researchers defined passion as “a state of profound physiological arousal” (Hatfield and Walster, 1978), “a drive that lead to similar phenomena like romance or physical attraction in a close relationship” (Sternberg, 1986), and “exceptionally strong feelings toward one’s partner” (Baumeister and Bratslavsky, 1999). Unlike intimacy, which takes a certain amount of time to develop, passionate feelings can emerge quickly at the beginning stages of a relationship; however, they tend to decline as time passes (Sternberg, 1986; Acker and Davis, 1992; Beutel et al., 2008).

Several studies show that intimacy and passion are positively associated (e.g., Sangrador and Yela, 2000; Weigel, 2010; Graham, 2011). Nevertheless, the causal direction of this association is not clear. Some studies suggest that intimacy predicts passion (Aron and Aron, 1986; Reissman et al., 1993; Aron et al., 2000; O’Leary et al., 2011; Birnbaum et al., 2016), whereas others suggest that passion predicts intimacy (Ratelle et al., 2013). The current study focuses on a model which proposes increases in intimacy generate passion (Baumeister and Bratslavsky, 1999).

### Passion as a Function of Change in Intimacy

The intimacy change model (Baumeister and Bratslavsky, 1999) is represented in mathematical terms as *P = (C) f(dI/dt)*. In this formula, *P* represents passion, *I* represents intimacy, *t* represents time, and *C* represents a constant that accounts for other factors that could moderate the required amount of increase in intimacy to generate passion. According to the model, increases in intimacy generate passion, and when intimacy levels remain stable no passion is generated. Thus, during the early stages of a relationship when intimacy is increasing steadily, passion levels will be high. However, intimacy will eventually reach its peak and remain stable, resulting in low levels of passion. This model provides a parsimonious framework that can explain the relationship between intimacy and passion, as well as, their time course.

To date, there has been only one empirical study that tested the intimacy change model. In a 21-day diary study with 67 couples it was found that increases in intimacy significantly predicted passion (Rubin and Campbell, 2012). Researchers also examined the partner effects (i.e., whether increases in one’s intimacy level increases partner’s passion) and found that increases in partner’s daily intimacy also positively predicted one’s daily passion. However, changes in passion also significantly predicted intimacy, but the authors suggested that the support for this opposite direction hypothesis was weaker. Similarly, we hypothesized that increases in one’s intimacy would be associated positively with one’s passion (H1). Since the previous work also found support for opposite direction hypothesis (Rubin and Campbell, 2012), and there can be a bidirectional association between these two variables, we also tested whether increases in one’s passion would be associated positively with one’s intimacy as an alternative model.

Researchers also suggested that intimacy should be conceptualized in an interpersonal framework as it includes mutual self-disclosure, trust, and communication (Reis and Patrick, 1996; Ferreira et al., 2012; Rubin and Campbell, 2012). One’s self-disclosure in the relationship may not be enough to generate intimacy, because partner’s warm and positive reactions to self-disclosure also play an important role in intimacy development (Reis et al., 2004). Therefore, we also examined the association between one’s partner’s intimacy change and one’s own passion. We hypothesized that increases in partner’s intimacy would be associated positively with one’s passion, independent of one’s own increases in intimacy (H2). As an alternative model, we also tested whether increases in partner’s passion would be associated positively with one’s intimacy, independent of one’s own increases in passion (Rubin and Campbell, 2012).

## Materials and Methods

### Participants

Both partners of 75 heterosexual couples who had been in a romantic relationship for at least 1 month participated in the study. Participants were recruited from undergraduate courses at a large university. Students received extra course credit for their participation in the study. Data from one couple were not included in the analysis because the couple did not follow the instructions. All the couples were in a dating relationship, and three couples were living together. The average age of the participants was 21.35 years (*SD* = 1.88), and the length of the relationships ranged between 1 and 124 months (*M* = 21.66, *SD* = 21.88).

### Measures

#### Person-Level Measures

##### Intimacy

Person-level intimacy was measured by 15 items from the Sternberg’s Triangular Love Scale (Sternberg, 1988). Participants rated items such as “I have a warm relationship with my partner” and “I feel emotionally close to my partner” on a 1 (*strongly disagree*) to 7 (*strongly agree*) scale. Internal reliability was 0.90.

##### Passion

Person-level passion was measured by 15 items from the Sternberg’s Triangular Love Scale (Sternberg, 1988). Participants rated items such as “I cannot imagine life without my partner” and “I adore my partner” on a 1 (*strongly disagree*) to 7 (*strongly agree*) scale. Internal reliability was 0.93.

##### Commitment

Person-level commitment was measured by 15 items from the Sternberg’s Triangular Love Scale (Sternberg, 1988). Participants rated items such as “I am committed to maintaining my relationship with my partner” and “I view my relationship with my partner as permanent” on a 1 (*strongly disagree*) to 7 (*strongly agree*) scale. Internal reliability was 0.94. This measure was used for measurement validity, and it was not included in models testing main hypothesis.

#### Day-Level Measures

##### Intimacy

Day-level intimacy was measured using four items from the Intimacy Scale (Rubin and Campbell, 2012). One item measured self-disclosure (“Today, I disclosed my thoughts and emotions”), two items measured favorable attitude (“I felt a lot of closeness and intimacy today”; “Our relationship was close today”), and one item measured communication of affection (“Today, I was affectionate with my partner”). Participants rated the items on a 1 (*strongly disagree*) to 7 (*strongly agree*) scale. Before daily alphas were computed, items were within-person centered to remove the between-person effects from the ratings. Daily alphas ranged from 0.87 to 0.97, and the mean alpha across the 14-day period was 0.94.

##### Passion

Day-level passion was measured using three items, one item from Hatfield and Walster’s (1978) definition of passion (“To what extent did you want your partner beside you today?”), one item from Sternberg’s (1988) Triangular Love Scale (“How often did you think about your partner today?”), and one item from Rubin and Campbell’s (2012) study (“How passionate was your relationship today?”). Participants rated the items on a 1 (*not at all*) to 7 (*extremely*) scale. Before daily alphas were computed, items were within-person centered to remove the between-person effects from the ratings. Daily alphas ranged from 0.64 to 0.86, and the mean alpha across the 14-day period was 0.78.

### Procedure

The study involved three phases: an orientation session, an online questionnaire packet, and 14 days of interval-contingent diary records. Initially, participants were invited to the lab to receive an orientation session on how to complete the daily records. Participants were instructed to complete the records every day before going to bed. The researcher also explained the importance of completing the records independently from each other and told participants to keep their responses private.

After the orientation session, each participant received an e-mail providing a link for the baseline questionnaire packet that included demographics and person-level measures^1^. To ensure that participants had sufficient time to complete the questionnaires, they were given 1 week before they proceeded to the diary phase of the study.

During the diary phase, participants submitted an online record at the end of each day for 14 consecutive days. Those who forgot to complete a day’s record received a reminder e-mail the following morning, and they were allowed to submit their records until noon of the following day. The time-stamped data were checked to confirm that the participants followed the instructions and completed the records on-time. The average number of completed records was 13.10 (*SD* = 1,41) for males and 13.46 (*SD* = 1,02) for females. Two participants completed an extra record and these records were excluded from the analysis.

### Data Analytic Strategy

The data structure was hierarchically nested as two individuals were nested within 74 couples that were then crossed with 14 days. Because the data consisted of distinguishable dyads, we used a multilevel model with two-intercepts (Bolger and Shrout, 2007) to adjust for non-independence. The two-intercept model approach allows separate estimates for females and males. The female dummy-coded variable (1 for females and 0 for males) and the male dummy coded variable (1 for males and 0 for females) are multiplied by a predictor to have two separate estimates for females and males. Therefore, we reported the separate estimates for each gender using *bf* for females and *bm* for males.

We used the PROC MIXED routine with maximum likelihood estimation in SAS Software to estimate the coefficients. Furthermore, the first-order autoregressive covariance structure type (AR) was specified to model the correlation between one’s daily outcome (e.g., passion) and the outcome that immediately preceded it (one’s passion from the day before). This structure allowed the errors to be auto correlated to model the correlation from 1 day to the next (Kenny et al., 2006). In all of the analyses, day-level predictor variables were within-person centered, and person-level predictors (intimacy or passion) were controlled. All of the regression coefficients reported in multilevel analyses are unstandardized.

## Results

### Results for Measurement Validity

To establish measurement validity for person-level measures, we conducted a confirmatory factory analysis for the person-level triangular love scale, and compared the three factor (intimacy, passion, commitment) structure with one-factor (love) structure. Results showed better fit indices for three-factor structure [χ^2^(990) = 5628.20, *p* < 0.001, 90% CI [0.10, 0.11], CFI = 0.71, RMSEA = 0.10], compared to the one-factor structure [χ^2^(990) = 5628.20, *p* < 0.001, 90% CI [0.11, 0.12], CFI = 0.62, RMSEA = 0.12]. We also conducted an exploratory principal component factor analysis. Results mainly showed a three factor pattern. Out of 15 intimacy items, two of them mainly loaded on the other factors, and another two items cross-loaded on the other factors. Out of 15 passion items, two of them mainly loaded on the other factors.

Next, we conducted confirmatory multilevel factor analyses to establish measurement validity for day-level measures. We created a single factor latent variable model with seven indicators, and a two factor model using four intimacy and three passion items. Although neither of the models showed good fit, the two factor model showed a better fit CFI = 0.825, RMSEA = 0.091, SRMR_within_ = 0.048 with a good fit for the within level, compared to the single factor model CFI = 0.650, RMSEA = 0.123, SRMR_within_ = 0.08. However, these findings might not be robust because the sample size was small compared to the number of parameters estimated in the model.

We also conducted additional analyses using the other variables in the dataset as outcome variables to examine discriminant validity. First, we had one item measuring whether people wanted to express their love to their partners using physical contact expressions. We expected that this variable should be more closely related to passion rather than intimacy. Passion significantly predicted physical contact in males (*b* = 0.66, *p* < 0.001) and intimacy did not (*b* = 0.27, *p* = 0.32). For females, both passion (*b* = 0.53, *p* < 0.001) and intimacy (*b* = 0.43, *p* = 0.029) significantly predicted physical contact. Second, we examined whether these variables predicted secrecy from partner. As intimacy is closely related to self-disclosure, we expected that intimacy, compared to passion, should be a stronger predictor of secrecy. The association of intimacy with secrecy was (*b* = -0.26, *p* = 0.21) for females, (*b* = -0.21, *p* = 0.20) for males. The association of passion with secrecy was (*b* = -0.14, *p* = 0.46) for females, (*b* = -0.03, *p* = 0.82) for males. Third, we conducted the same analysis for trust in partner. Trust is associated with self-disclosure; thus it should also be more strongly predicted by intimacy. For females, intimacy significantly predicted trust (*b* = 0.37, *p* = 0.03) but passion did not (*b* = 0.19, *p* = 0.10). For males, neither intimacy (*b* = 0.38, *p* = 0.15) nor passion (*b* = 0.24, *p* = 0.15) significantly predicted trust. These findings show that intimacy measure was more strongly related to intimacy related constructs, whereas passion measure was more strongly related to physical contact.

### Results for Main Hypothesis

In order to examine whether changes in intimacy were associated with daily passion (H1), we created residualized intimacy change scores by regressing the current day’s intimacy on the previous day’s intimacy, following the analysis steps of Rubin and Campbell (2012). We used a model with two random intercepts, two random slopes, and person-level intimacy as a control variable. Results showed that person-level intimacy was associated positively with daily passion for both females and males (*bf* = 0.80, *SE* = 0.13, *p* < 0.001, 95% CI [0.55, 1.06], *bm* = 0.53, *SE* = 0.13, *p* < 0.001, 95% CI [0.28, 0.79]). Also, changes in daily intimacy positively predicted daily passion for both females and males (*bf* = 0.39, *SE* = 0.02, *p* < 0.001, 95% CI [0.33, 0.45], *bm* = 0.34, *SE* = 0.03, *p* < 0.001, 95% CI [0.29, 0.41]). The findings are summarized in **Table 1**. The results indicated that increases in intimacy predicted greater passion for both males and females.

**Table 1:** Intimacy change predicting passion.

Variable	Parameter estimate	*SE*	*t*-value	95% CI [lower, upper]	Random effects
Intercept F	0.17	0.82	0.21	[-1.47, 1.81]	0.46^∗∗∗^
Intercept M	2.12^∗^	0.81	2.61	[0.50, 3.74]	0.54^∗∗∗^
Prsn Lvl Intm. F	0.80^∗∗∗^	0.13	6.28	[0.55, 1.06]	–
Prsn Lvl Intm. M	0.53^∗∗∗^	0.13	4.16	[0.28, 0.79]	–
Intimacy Chn. F	0.39^∗∗∗^	0.02	18.04	[0.33, 0.45]	0.01^∗^
Intimacy Chn. M	0.34^∗∗∗^	0.03	12.96	[0.29, 0.41]	0.02^∗^

^∗^*p < 0.05, ^∗∗^*p* < 0.01, ^∗∗∗^*p* < 0.001.*

*Random effects represent random intercept and random slope variances*.

We also examined the alternative hypothesis by testing whether changes in passion predict intimacy (passion change model). We used the same analysis approach with residualized daily passion scores along with person-level passion as predictors of daily intimacy. Results showed that person-level passion was associated positively with daily intimacy only for females (*bf* = 0.29, *SE* = 0.07, *p* < 0.001, 95% CI [0.17, 0.45], *bm* = 0.07, *SE* = 0.10, *p* = 0.50, 95% CI [-0.14, 0.26]). However, changes in daily passion positively predicted daily intimacy for both females and males (*bf* = 0.50, *SE* = 0.04, *p* < 0.001, 95% CI [0.42, 0.58], *bm* = 0.50, *SE* = 0.04, *p* < 0.001, 95% CI [0.42, 0.58]) (see **Table 2**).

**Table 2:** Passion change predicting intimacy.

Variable	Parameter estimate	*SE*	*t*-value	95% CI [lower, upper]	Random effects
Intercept F	3.92^∗∗∗^	0.40	9.74	[3.12, 4.72]	0.44^∗∗∗^
Intercept M	4.95^∗∗∗^	0.58	8.50	[3.80, 6.11]	1.07^∗∗∗^
Prsn Lvl Pass. F	0.29^∗∗∗^	0.07	4.05	[0.17, 0.45]	–
Prsn Lvl Pass. M	0.07	0.10	0.68	[-0.14, 0.26]	–
Passion Chn. F	0.50^∗∗∗^	0.04	14.37	[0.42, 0.58]	0.03^∗^
Passion Chn. M	0.50^∗∗∗^	0.04	11.67	[0.41, 0.58]	0.05^∗∗^

^∗^*p < 0.05, ^∗∗^*p* < 0.01, ^∗∗∗^*p* < 0.001.*

*Random effects represent random intercept and random slope variances*.

### Partner Effects

Next, we examined whether partner’s intimacy change was associated with one’s own passion (H2), using similar sets of analyses. Actor and partner effects were estimated in the same step to examine the unique effect of each in predicting the criterion. An actor effect implies that one’s own intimacy change predicts one’s own passion, independent of one’s partner’s intimacy change. A partner effect means that one’s partner’s intimacy change predicts one’s own passion, independent of one’s own intimacy change (APIM; Kashy and Kenny, 2000; Campbell and Kashy, 2002). In order to test the partner effect hypothesis, we first created residualized change scores for partner’s intimacy and conducted a multilevel analysis with actor’s intimacy change, partner’s intimacy change as predictors of actor’s passion. Results showed that both actor’s and partner’s daily intimacy change uniquely predicted actor’s daily passion. Partner’s daily intimacy change was associated positively with actor’s daily passion (*bf* = 0.09, *SE* = 0.02, *p* < 0.001, 95% CI [0.05, 0.13], *bm* = 0.09, *SE* = 0.02, *p* < 0.001, 95% CI [0.05, 0.13]), independent of actor’s daily intimacy change (*bf* = 0.37, *SE* = 0.02, *p* < 0.001, 95% CI [0.33, 0.41], *bm* = 0.31, *SE* = 0.03, *p* < 0.001, 95% CI [0.24, 0.36]). These findings (**Table 3**) showed that individuals reported greater passion when their partners reported greater increases in their intimacy, independent from increases in their own intimacy.

**Table 3:** Actor and partner intimacy change predicting passion.

Variable	Parameter estimate	*SE*	*t*-value	95% CI [lower, upper]	Random effects
Intercept F	-0.01	0.82	-0.01	[-1.65, 1.63]	0.44^∗∗∗^
Intercept M	2.07^∗^	0.81	2.56	[0.45, 3.69]	0.51^∗∗∗^
Prsn Lvl Intm F	0.83^∗∗∗^	0.13	6.55	[0.57, 1.09]	-
Prsn Lvl Intm M	0.55^∗∗∗^	0.13	4.24	[0.28, 0.80]	-
A. Intm. Chn. F	0.37^∗∗∗^	0.02	15.01	[0.33, 0.41]	0.01^∗^
A. Intm. Chn. M	0.31^∗∗∗^	0.03	10.77	[0.25, 0.37]	0.02^∗∗^
P. Intm. Chn. F	0.09^∗∗∗^	0.02	3.97	[0.05, 0.13]	–
P. Intm. Chn. M	0.09^∗∗∗^	0.02	4.27	[0.05, 0.13]	–

^∗^*p < 0.05, ^∗∗^*p* < 0.01, ^∗∗∗^*p* < 0.001.*

*Random effects represent random intercept and random slope variances. There were no random slopes defined in the model for the partner effects*.

We also tested the partner effects for the passion change model. We used residualized change scores of passion for both actor and partner as predictors of actor’s intimacy. Results showed that changes in partner’s daily passion positively predicted actor’s daily intimacy (*bf* = 0.12, *SE* = 0.03, *p* = 0.001, 95% CI [0.05, 0.17], *bm* = 0.21, *SE* = 0.03, *p* < 0.001, 95% CI [0.15, 0.27]), independent of changes in actor’s daily passion (*bf* = 0.52, *SE* = 0.04, *p* < 0.001, 95% CI [0.45, 0.61], *bm* = 0.44, *SE* = 0.04, *p* < 0.001, 95% CI [0.36, 0.52]). The findings are presented in **Table 4**. Overall, these results indicated that individuals reported greater intimacy when their partners reported greater increases in their passion independent from increases in their own passion.^2^

**Table 4:** Actor and partner passion change predicting intimacy.

Variable	Parameter estimate	*SE*	*t*-value	95% CI [lower, upper]	Random effects
Intercept F	3.89^∗∗∗^	0.39	9.75	[3.09, 4.67]	0.40^∗∗∗^
Intercept M	4.79^∗∗∗^	0.57	8.40	[3.65, 5.93]	0.97^∗∗∗^
Prsn Lvl Pass. F	0.30^∗∗∗^	0.07	4.18	[0.16, 0.44]	–
Prsn Lvl Pass. M	0.10	0.10	1.02	[–0.10, 0.30]	–
A. Pass. Chn. F	0.52^∗∗∗^	0.04	13.75	[0.45, 0.61]	0.02
A. Pass. Chn. M	0.44^∗∗∗^	0.04	10.63	[0.36, 0.52]	0.04^∗∗^
P. Pass. Chn. F	0.12^∗∗^	0.03	3.57	[0.06, 0.18]	–
P. Pass. Chn. M	0.21^∗∗∗^	0.03	6.97	[0.15, 0.27]	–

^∗^*p < 0.05, ^∗∗^*p* < 0.01, ^∗∗∗^*p* < 0.001.*

*Random effects represent random intercept and random slope variances. There were no random slopes defined in the model for the partner effects*.

**FIGURE 1:**
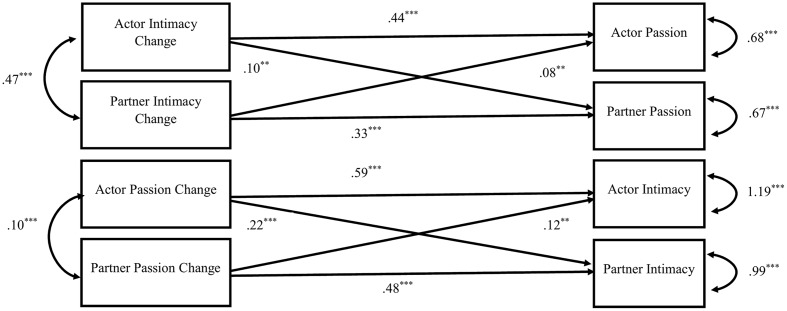
Within person effects. Values represent unstandardized parameter estimates. ^∗^*p* < 0.05, ^∗∗^*p* < 0.01, ^∗∗∗^*p* < 0.001.

**FIGURE 2:**
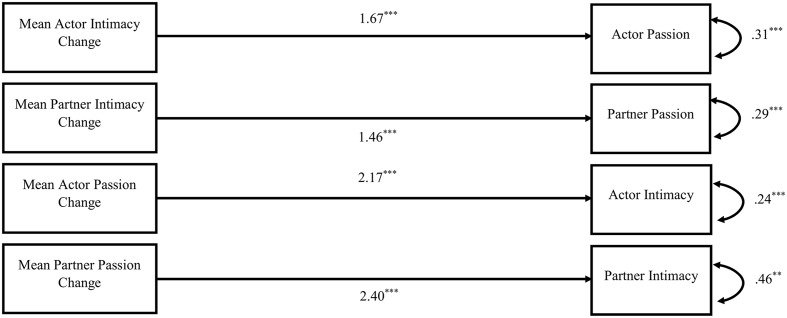
Between person effects. Values represent unstandardized parameter estimates. ^∗^*p* < 0.05, ^∗∗^*p* < 0.01, ^∗∗∗^*p* < 0.001.

Finally, we conducted a multilevel path analysis to test all of the associations simultaneously. We used a multilevel APIM structure to examine these associations. The dyads were distinguishable by gender, thus we coded females as actors and males as partners. Non-independence between couples was modeled with covariances between actor and partner variables. Residualized change scores were centered within individuals by subtracting each actor’s or partner’s mean score across 14 days from their daily scores. Moreover, we used the mean scores across 14 days as the person-level variables, instead of the scores from the triangular love-scale. The findings for within-person and between-person associations are shown in **Figures 1**, **2**. Within-person associations were similar to that of two-intercept models, with significant actor and partner effects for both intimacy change and passion change. Between-person results showed significant effects for both intimacy change and passion change, but there were no significant partner effects.

## Discussion

The present study provides a further test of the intimacy change model (Baumeister and Bratslavsky, 1999), which has been examined empirically only in one study (Rubin and Campbell, 2012). Although we replicated the main findings of Rubin and Campbell (2012), our analyses also support the idea that changes in passion predict intimacy. Thus, there was no unequivocal support for the intimacy change model, both models were supported.

Rubin and Campbell (2012) found weaker support for the passion change hypothesis compared to the intimacy change hypothesis; however, they also argued that the relationship between the two could be reciprocal. Our analyses using similar methods also suggest that both models are viable, and that the relationship between the two might be reciprocal. In sum, the findings suggest that the tested model (Baumeister and Bratslavsky, 1999) may not be sufficient to explain the association between intimacy and passion.

We also explored partner effects in intimacy change and passion relationship and found significant partner effects for both models. Moreover, multilevel path analysis also showed similar significant effects, when both models and partner effects were tested simultaneously. This finding shows the dynamic nature of the association between intimacy and passion, as changes in partner’s intimacy or passion can have a unique effect on one’s own intimacy or passion. Thus, the association between intimacy and passion seems to be more complex than hypothesized, and researchers should focus on dyadic processes to further our understanding of these processes.

More important, future studies may also emphasize establishing the difference between intimacy and passion. The current study tested a model that was based on the assumption that intimacy and passion are separate dimensions of love. Some researchers raised concerns on the triangular model and Sternberg’s Triangular Love Scale, as there is high overlap between these dimensions (Acker and Davis, 1992; Graham, 2011). However, our factor analysis with the person-level triangular love-scale showed that passion and intimacy are two-separate factors. Furthermore, we also conducted further multilevel analyses with the daily items, which provided some support that these items measured different constructs. Nevertheless, the limited number of items used in the diary records do not allow us to make unequivocal conclusions about the process between intimacy and passion. Future studies that focus on whether intimacy and passion are actually separate aspects of love, or studies that provide improved measures of these constructs with larger sample sizes are needed before examining the seemingly complex process between intimacy and passion.

There are also other caveats of the study that should be noted. First, residualized scores involve both previous day’s score and current day’s score. Thus, the significant effect of these change scores also involves a combination of both of these variables. More advanced statistical methods, such as differential models, might be more appropriate for testing the model. Second, our findings are still correlational, which do not allow drawing conclusions about causality.

The current findings provided some empirical evidence for the hypothesis that increases in intimacy generates passion (Baumeister and Bratslavsky, 1999), in line with the previous research (Rubin and Campbell, 2012). However, our results also suggest that it is not possible to discern whether increases in intimacy generates passion or increases in passion generates intimacy. Moreover, partner’s passion and intimacy change also seems to be important for one’s own intimacy and passion. In brief, the intimacy change model does not appear to adequately explain the complex process between intimacy and passion.

## Ethics Statement

This study was carried out in accordance with the recommendations of ‘Applied Ethics Research Center of Middle East Technical University’ with written informed consent from all subjects. All subjects gave written informed consent in accordance with the Declaration of Helsinki.

## Author Contributions

BA and AU made contributions on conception and design of study, acquisition of data, analysis and/or interpretation of data, and drafting the manuscript.

## Conflict of Interest Statement

The authors declare that the research was conducted in the absence of any commercial or financial relationships that could be construed as a potential conflict of interest.

## Footnotes

1 The person-level questionnaire also included measures of life satisfaction, self-concealment, trust, jealousy, need satisfaction, conflict, attachment, agreeableness, openness to experience, conscientiousness, and neuroticism. The diary records also included items measuring perceived intimacy, physical attractiveness, relationship satisfaction, conflict, life satisfaction, trust, need satisfaction, self-concealment, jealousy, and emotion expression.

2 Baumeister and Bratslavsky (1999) suggested that relationship length and extraversion may moderate the relationship between intimacy and passion. Thus, we also examined the moderating effects of these variables. However, there was no main effect (*bf* = 0.00, *p* = 0.51, *bm* = -0.00, *p* = 0.37), and no significant interaction effect of relationship length (*bf* = 0.00, *p* = 0.16, *bm* = -0.00, *p* = 0.57). As for extraversion, there was a negative main effect for females (*bf* = -0.24, *SE* = 0.11, *p* = 0.035, 95% CI [-0.46, -0.02], *bm* = 0.06, *SE* = 0.12, *p* = 0.61, 95% CI [-0.18, 0.30]), but no interaction effect (*bf* = 0.02, *SE* = 0.03, *p* = 0.53, 95% CI [-0.04, 0.08], *bm* = -0.02, *SE* = 0.03, *p* = 0.46, 95% CI [-0.08, 0.04]).
